# Urbanization, Not Invasive Shrubs, Alters Tree Seed Fate by Modifying Rodent Activity

**DOI:** 10.1002/ece3.72038

**Published:** 2025-08-22

**Authors:** Mark E. Fuka, Brian M. Connolly, John L. Orrock

**Affiliations:** ^1^ University of Wisconsin‐Madison Madison Wisconsin USA; ^2^ Gonzaga University Spokane Washington USA

**Keywords:** animal activity, *Rhamnus cathartica*, seasonality, seed removal, urbanization

## Abstract

Changes in the global environment are widespread and may have unappreciated effects on the activity of animals and the strength of animal‐mediated interactions. For example, urbanization and the spread of invasive species are aspects of global change that may lead to shifts in the activity of granivorous rodents, potentially leading to changes in the survival and establishment of seeds rodents consume. Importantly, these two aspects of global change could interact to affect rodent activity. We used a large‐scale manipulation of common invasive shrubs ( *Rhamnus cathartica* , *Lonicera macckii*) across a rural‐to‐urban forest gradient spanning southern Wisconsin in summer and autumn to examine the effects that urbanization, invasion, and seasonality had on modifying rodent activity and granivory. Using two 14‐day sampling sessions, we recorded the activity of three granivorous rodents ( *Peromyscus leucopus* , *Sciurus carolinensis* , and *Tamias striatus* ) with motion‐activated cameras and quantified seed removal using six tree species ( *Quercus rubra* , *Quercus alba* , *Acer saccharum* , *Prunus serotina* , *Pinus strobus* , and *Pinus resinosa* ) with seed depots to link animal activity with seed removal. Our findings reveal that *Quercus* seed removal was highest in urban sites, potentially linking hotspots of *S. carolinensis* activity in urban forests to decreased seed survival. In capturing a large number of *P. leucopus* photos during the autumn in *R. cathartica* removed plots, our findings suggest that *P. leucopus* may be responding to the provision of short‐term resources. Our results underscore the primacy of seed mass in determining rodent seed choice: although urbanization and invasive shrubs had different effects on the activity of rodent species, the removal of seeds was always strongly dependent upon seed mass. Our findings help to illuminate potential hotspots of granivorous rodent activity along an urbanization gradient, the shifts in species‐specific seed loss associated with this gradient, and the dominance of seed mass in contributing to rodent seed preference.

## Graphical abstract

Urbanization and the spread of invasive species are aspects of global change that may lead to shifts in the activity of granivorous rodents, potentially leading to changes in the survival and establishment of seeds rodents consume. Our findings help to illuminate potential hotspots of granivorous rodent activity along an urbanization gradient, the shifts in species‐specific seed loss associated with this gradient, and the dominance of seed mass in contributing to rodent seed preference.

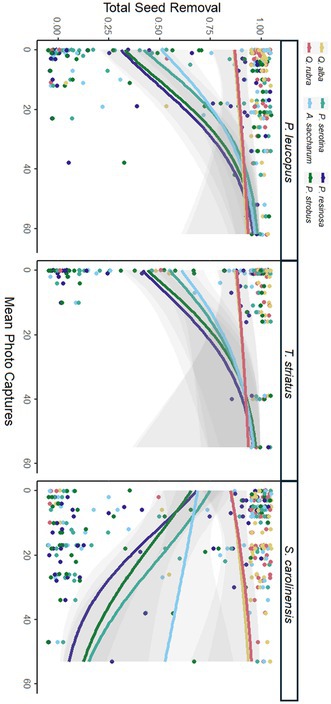

## 1 Introduction

Novel environments created by global change (e.g., introduction of non‐native species, land conversion) can alter animal behavior, activity, and community composition (Robertson et al. 2013; Wong and Candolin 2015; Wilson et al. 2020). Invasive plants, for example, are common within many ecosystems, and these species alter spatial and temporal patterns of animal activity (Ceradini and Chalfoun 2017; Guiden and Orrock 2019; Connolly et al. 2024; Stewart et al. 2021). Additionally, urbanization can affect animal communities by altering predator abundances that shape urban trophic dynamics and by modifying animal behavior given anthropogenic activity and food subsidies (Fischer et al. 2012; Persons and Eason 2017; Gallo et al. 2019; Potgieter et al. 2024). Importantly, although animals may experience both invasive species and urbanized environments (Shochat et al. 2010; Persons and Eason 2017; Borden and Flory 2021), it is not known whether these two aspects of global change (invasive species and urbanization) might interact to affect animal activity. For example, although invasive shrubs may modify rodent activity by reducing perceived predation risk (Mattos et al. 2013; Guiden and Orrock 2019), these changes in activity might be offset by responses to changes in the sound and light environment associated with urbanization (Lyons et al. 2017; Gallo et al. 2022; Tranquillo et al. 2024). Understanding how invasive species and urbanization might act, alone or in concert, to affect animal activity is important for understanding patterns of animal distribution and abundance, but also for understanding variation in the strength of animal‐mediated interactions.

Seed removal by granivorous rodents is an interaction with the potential to limit the distribution and abundance of plants (Howe and Brown 2001; Crawley 2013; Larios et al. 2017; Dylewski et al. 2020). Seed predation can vary significantly in space and time (Plucinski and Hunter Jr. 2001), and previous work suggests that both invasive species and urbanization may play a role in determining trends in small mammal activity and the removal of seeds by small mammal granivores. Invasive woody shrubs (e.g., common buckthorn, *Rhamnus cathartica* , and amur honeysuckle, *Lonicera maackii* ) provide shelter from avian predators and produce food resources that can lead to changes in rodent (e.g., *Peromyscus* spp.) anti‐predator behavior (Mattos and Orrock 2010; Guiden and Orrock 2020, 2019). Additionally, invasive shrubs can increase activity in invaded habitats (Dutra et al. 2011; Guiden and Orrock 2019) and create seasonally available habitat structure that can modify seasonal patterns of seed removal (Bartowitz and Orrock 2016; Guiden and Orrock 2019; Fuka and Orrock 2024). Urbanization can lead to changes in the timing and amount of small‐mammal activity (Gallo et al. 2022; Burton et al. 2024), and rodents in urban forests may also display differences in vigilance and foraging behaviors (Mazza et al. 2020; Tranquillo et al. 2024) that could affect seed removal. Despite the potential for invasive shrubs and urbanization to affect rodent activity and granivory, the effect of these two aspects of global change remains unexamined.

Using a large‐scale manipulation of invasive shrub cover at 15 forested sites spanning a rural‐to‐urban gradient, we examined the effect of urbanization and invasive woody shrubs on seasonal patterns of small mammal activity and seed removal. Common buckthorn and amur honeysuckle are introduced shrub species prevalent throughout eastern North American forests; both shrub species modify habitat structure and provide seasonal resources that have been shown to modify small mammal activity and granivory (Knight et al. 2007; Mattos et al. 2013; Guiden and Orrock 2019; Schuster et al. 2020). We focus our study on the activity of three common small mammal species: the white‐footed mouse ( *Peromyscus leucopus* ), eastern chipmunk ( *Tamias striatus* ), and eastern gray squirrel ( *Sciurus carolinensis* ), as other studies demonstrate these small mammal species play a significant role in the removal and consumption of the *Quercus* spp., *Pinus* spp., and *Acer* spp. seeds in oak‐pine temperate U.S. forests (e.g., Plucinski and Hunter Jr. 2001). We paired activity density estimates with concurrent measures of seed removal on six dominant tree species native to upper midwestern United States forests, that is, white oak ( *Quercus alba* ), red oak ( *Quercus rubra* ), sugar maple ( *Acer saccharum* ), black cherry ( *Prunus serotina* ), white pine ( *Pinus strobus* ), and red pine ( *Pinus resinosa* ). We predicted that (1) greater rodent activity, amplified by invasive shrubs, would correspond to greater seed removal; (2) elevated risk‐taking and exploratory behaviors associated with urban‐dwelling rodents (Mazza et al. 2020; Tranquillo et al. 2024) would lead to higher animal activity in urban than in rural forests; and (3) large, often desirable seeds (e.g., *Quercus* spp.) would be taken at greater rates (Radtke 2011; Dylewski et al. 2020) than smaller seeds (e.g., *Pinus* spp.).

## 2 Methods

### 2.1 Study Location and Plot Design

To quantify rodent activity and seed removal across a rural‐to‐urban forest gradient, we used 15 forested sites across Southern Wisconsin spanning from Madison, WI, to Milwaukee, WI (Appendix S1, Figure S1). Each site was dominated by invasive woody shrubs throughout the understory (primarily *R. cathartica* with a fraction of *L. macckii*) with similar canopy openness and topography (Appendix S1). Common overstory tree species consisted of *P. serotina* , *A. saccharum* , and *Quercus* spp. (primarily red and white oak). Using land cover data downloaded from the National Land Cover Database (Dewitz 2023), forests were chosen based on varying levels of surrounding human development (Appendix S1). We selected sites with moderate‐to‐high, homogeneous invasive shrub stem density to best measure the effects of shrub management (mean: 7.2 stems/m^2^). To capture any potential effects that surrounding development may have on small mammal activity, we used a 250 m buffer from each site when classifying land cover. This buffer ensured an area that could be traversed by each of our target small mammal species while still being large enough to capture differences in the level of surrounding development around each forest (López‐Barrera et al. 2005; Hämäläinen et al. 2019). Within each selected forest, 20 m from the edge, two adjacent 400 m^2^ plots were delineated, separated by 20 m. In each pair of plots, one plot was randomly chosen to have all invasive shrubs within it removed. Shrub removal was done using the “cut‐stump” method (Schuster et al. 2022), where each shrub was severed at the base of the stump (< 0.1 m above ground level) and triclopyr ester (Garlon‐4) was immediately applied to the root collar to prevent subsequent resprouting. We used principal component analysis of land cover types around each site (Agriculture, Development Intensity, Forest, Open Water, Wetland, Other) to create a site gradient of rural‐to‐urban forests, a common approach used to characterize land cover (Kerr and Cihlar 2003; Cifaldi et al. 2004). The first PC axis (PC1) explained 70.18% of the variance in the surrounding land cover classes. This “Development PC” was strongly positively correlated with development in the surrounding landscape (Appendix S1, Table S1, and Figure S2), making it a suitable variable for quantifying landscape composition surrounding our study sites. The use of a continuous variable for an urbanization term rather than a discrete term (i.e., more surrounding development vs. less surrounding development) allows for a more fine‐grained analysis of landscape complexity and therefore provides a better representation of natural landscapes along a gradient of landscape contexts (Manley et al. 2009).

### 2.2 Small Mammal Activity

To measure the activity of small mammals, each plot center (*n* = 30) was equipped with an infrared, motion‐activated camera trap (Bushnell 16MP Trophy Cam HD; Bushnell Corporation, Overland Park, KS) < 0.5 m above ground level. Cameras were set to capture 3‐round bursts of photos at the highest sensitivity to ensure small mammal detection. For analyses, an interval of 5 min between photos was used to distinguish between different individuals (Meek et al. 2014; Randler and Kalb 2020). Camera traps were activated for two 14‐day sampling periods in July 2023 (summer session; 07/04–07/18) and November 2023 (autumn session; 11/14–11/28), totaling 420 trap days across all cameras and sites. The timing of sampling sessions corresponded to the availability of canopy cover (e.g., both native tree and invasive shrub cover in the summer, only invasive shrub cover in the autumn). Photos were scored for the detections of *P. leucopus* , *T. striatus* , and *S. carolinensis* . On the basis of previous studies and observations in our study system (Guiden and Orrock 2019; Keller and Orrock 2023), we are assuming all *Peromyscus* spp. to be *P. leucopus*.

### 2.3 Seed Removal

To measure the magnitude of seed removal by rodent granivores, seed removal depots were placed at each plot center. Seed depots are 3.78 L buckets with 5 cm^2^ openings allowing small mammal access that are commonly used in seed removal studies to accurately measure the rate and magnitude of seed loss (Bartowitz and Orrock 2016; Chandler et al. 2020; Fuka and Orrock 2024; Fuka et al. 2024). Depots contained 44 seeds of native tree species common in the canopy of our study system, placed atop a sand substrate. Specifically, within each depot, we placed 10 seeds each of *A. saccharum, P. serotina, P. strobus*, and *P. resinosa* , as well as 2 acorns each of *Q. alba* and *Q. rubra* . Seed quantities are consistent with previous seed removal studies (Bartowitz and Orrock 2016; Chandler et al. 2020; Keller and Orrock 2023; Fuka and Orrock 2024) and replicate realistic naturally occurring seed densities in temperate forest seedbanks (Leckie et al. 2000). The number of remaining seeds was counted once halfway through deployment, 7 days after the start of each session. Following each 14‐day deployment session paired with camera trap deployments, depots were collected and brought to the lab, where sand was sifted from each depot, and the remaining, intact seeds were collected and counted. Seed removal is assumed to be analogous to seed death because of evidence, suggesting that all moved seeds are typically destroyed (Guiden and Orrock 2020; Fuka et al. 2025) or remain in arboreal caches (Steele et al. 2001, 2011; Bartlow et al. 2018; Sachser et al. 2025).

### 2.4 Statistical Analysis

We estimated rodent activity density as the total number of rodent photos taken at each plot during each sampling session. We tested for differences in animal species‐specific activity density on the basis of invasion status ( *R. cathartica* invaded vs. *R. cathartica* removed), season (summer vs. autumn), animal species ( *P. leucopus* vs. *S. carolinensis* vs. *T. striatus* ), and surrounding human development (Development PC) by constructing a multilevel model (MLM) (package: “glmmTMB”; Brooks et al. 2017) with a negative binomial error distribution (Jackson et al. 2012; Bartel and Orrock 2023). To account for the split‐plot nature of our study design and repeated measures, we included the random effects of invasion status nested within site. To measure potential differences in seed removal within each depot, we constructed a MLM with invasion status ( *R. cathartica* invaded vs. *R. cathartica* removed), season (summer vs. autumn), and surrounding human development (Development PC) as explanatory variables with all possible interactions using a binomial error distribution with a logit link function. For both models, we included invasion status nested with the site as random effects to account for repeated measures and our split‐plot design. Using an MLM approach allowed for a complete investigation of whether animal activity density or seed removal depended on or was affected by the presence (or absence) of another species in conjunction with the primary predictor variables of invasion status, season, and surrounding human development. For both models, we also used likelihood ratio tests (LRT) to determine whether urbanization affected species‐specific mammal activity and seed removal by comparing full models to models without the random effect of urbanization. A significant random effect of the slope would indicate that species‐specific responses vary significantly with urbanization, and both models revealed a significant random effect of the slope. Because seed removal was measured on both days 7 and day 14, we measured the activity of small mammals between days 0–7 and days 8–14 using a generalized linear mixed model with time as a 2‐level factor representing the first and second half of the deployment, along with invasion status ( *R. cathartica* invaded vs. *R. cathartica* removed), season (summer vs. autumn), animal species ( *P. leucopus* vs. *S. carolinensis* vs. *T. striatus* ), and surrounding development (Development PC). All analyses were performed in R version 4.2.2 (R Core Team 2024).

## 3 Results

### 3.1 Animal Activity

**FIGURE 1:**
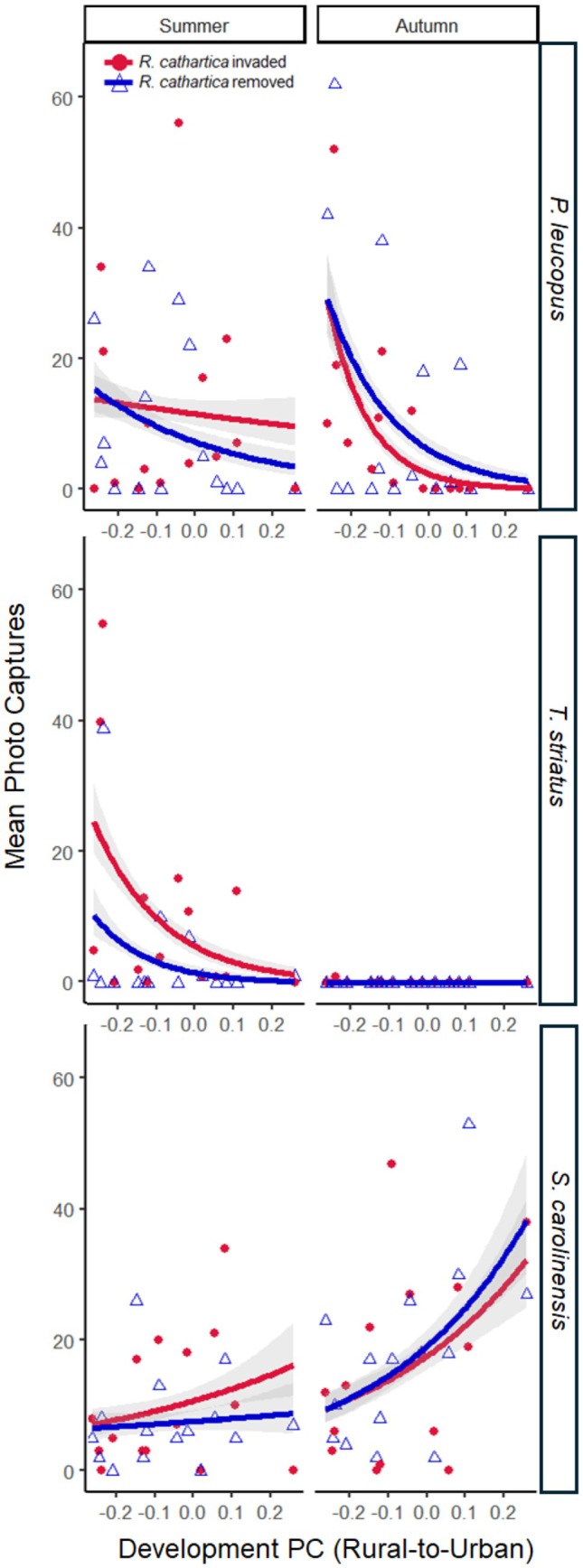
Mean photo captures (activity density) of *P. leucopus* , *T. striatus* , and *S. carolinensis* on the basis of invasion status ( *R. cathartica* invaded vs. *R. cathartica* removed) and season (summer vs. autumn) along the rural‐to‐urban forest gradient (Development PC).

A total of 1627 photos of small mammals (645 *P. leucopus* , 759 *S. carolinensis* , 223 *T. striatus* ) were captured across both sessions. Overall, small mammal activity was similar between seasons (*χ* ^2^ = 1.48, *p* = 0.222) and was not affected by invasion status (*χ* ^2^ = 1.63, *p* = 0.200) or surrounding human development (*χ* ^2^ = 0.57, *p* = 0.448) (Table 1). Examining mean slope estimates in the MLM indicated that among small mammal species, *S. carolinensis* was more active in urban forests than in rural forests (*β* = 1.51), whereas *P. leucopus* and *T. striatus* were less active in urban forests (*β* = −2.74, *β* = −2.33). *Tamias striatus* and *S. carolinensis* had similar levels of activity between *R. cathartica* invaded and *R. cathartica* removed plots during the deployment windows of days 1–7 and days 8–14, regardless of season. During the autumn, *P. leucopus* showed marginally higher activity in *R. cathartica* removal plots in the first half of the deployment than in the second half (*z* = −2.58, *p* = 0.092) (Figure 1).

**TABLE 1:** Results from the multilevel model examining the effects that invasion status ( *R. cathartica* invaded vs. *R. cathartica* removed), surrounding human development (Development PC), and season (summer vs. autumn) had on small mammal activity density.

Effect	ϐ	SE	*χ* ^2^	*p*
Invasion	−0.55	0.43	1.63	0.200
Development	−1.24	1.64	0.57	0.448
Season	−1.30	1.07	1.48	0.222
Invasion × development	−0.33	1.55	0.04	0.829
Invasion × season	0.44	0.41	1.13	0.286
Season × development	−2.36	1.85	1.62	0.202
Invasion × season × development	3.10	2.49	1.54	0.213

### 3.2 Seed Removal

**FIGURE 2:**
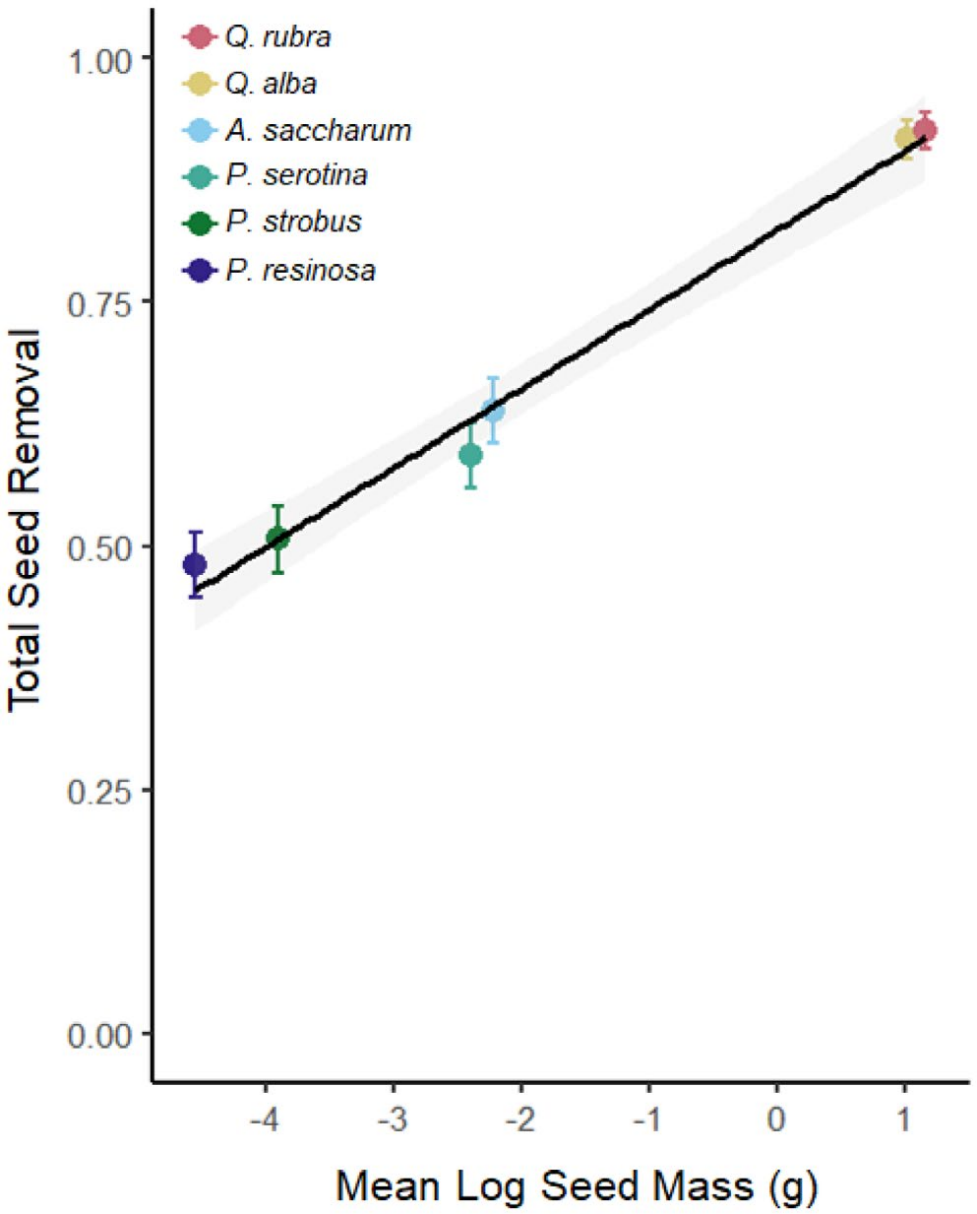
Proportion of species‐specific seed removal ( *Q. rubra* vs. *Q. alba* vs. *A. saccharum* vs. *P. serotina* vs. *P. strobus* vs. *P. resinosa* ) on the basis of average log seed mass (g).

**FIGURE 3:**
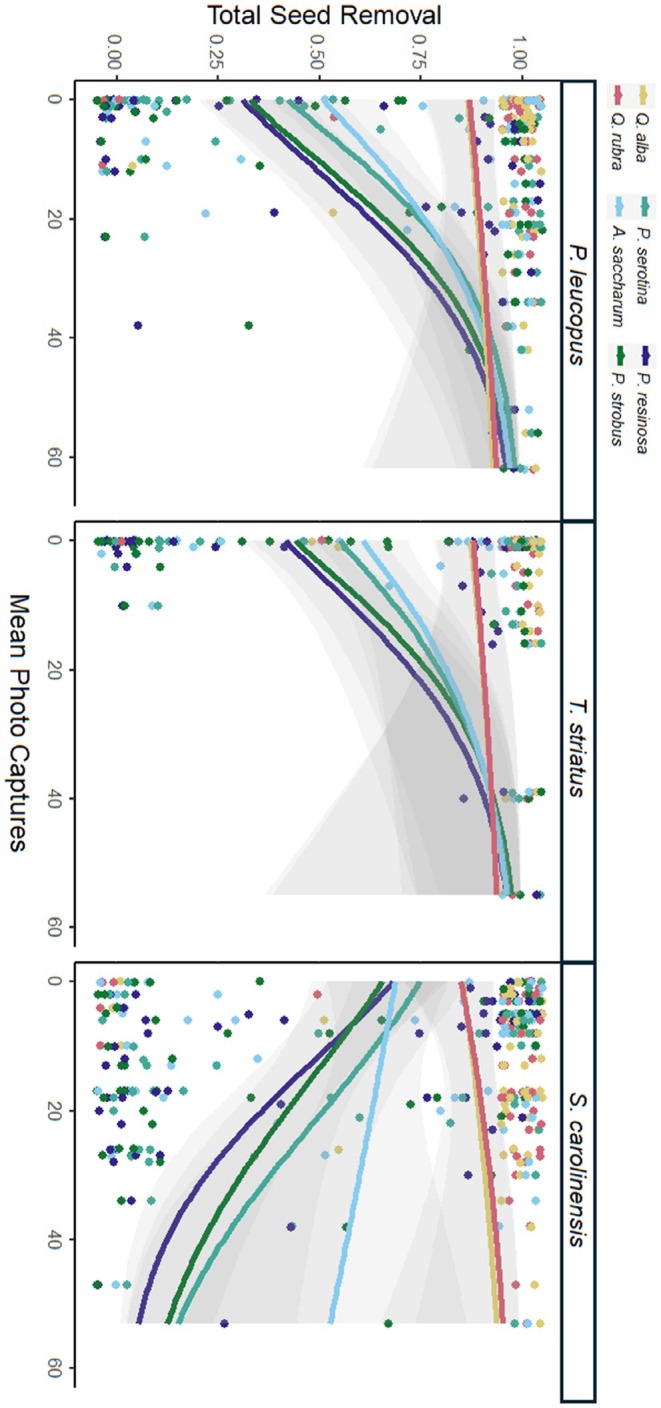
Proportion of species‐specific seed removal ( *Q. rubra* vs. *Q. alba* vs. *A. saccharum* vs. *P. serotina* vs. *P. strobus* vs. *P. resinosa* ) on the basis of mean photo captures (activity density) of *P. leucopus* , *T. striatus* , and *S. carolinensis*.

Of the 2640 seeds placed within all depots, a total of 1088 were removed across both sessions: 92.5% ± 3.3% *Q. rubra* , 91.6% ± 3.3% *Q. alba* , 63.8% ± 5.6% *A. saccharum* , 59.3% ± 5.7% *P. serotina* , 50.6% ± 5.8% *P. strobus* , and 48.0% ± 5.7% *P. resinosa* ; the proportion of seeds removed was strongly correlated with the individual seed mass (Figure 2; *r* ^2^ = 0.98, *p* < 0.001). Species‐specific tree seed removal did not differ by overall small‐mammal activity density (*χ* ^2^ = 1.18, *p* = 0.553), but increased *S. carolinensis* activity corresponded to lower *P. strobus* and *P. resinosa* removal than *Q. rubra* removal (*z* = 2.84, *p* = 0.051; *z* = −2.80, *p* = 0.057) (Figure 3). *Quercus rubra* and *Q. alba* were removed most in highly urban forests (*β* = 10.91, *β* = 10.21), whereas highly rural forests were most associated with *P. strobus* and *P. resinosa* seed removal (*β* = −3.44, *β* = −6.47) (Table 3). Both *Q. rubra* and *Q. alba* were removed at a significantly faster rate than *P. serotina* (*z* = −4.33, *p* < 0.001; *z* = −4.72, *p* < 0.001) and *A. saccharum* (*z* = 4.06, *p* < 0.001; *z* = −4.43, *p* < 0.001). Total seed removal was not affected by the presence of invasive shrubs (*χ* ^2^ = 0.01, *p* = 0.910) or surrounding human development (*χ* ^2^ = 0.10, *p* = 0.744), but was significantly higher in the summer (79.0% ± 5.4% removal) than in the autumn (56.3% ± 5.1% removal) (*χ* ^2^ = 7.94, *p* = 0.004) (Table 2), and this trend was driven by a large reduction in the removal of *P. serotina* , *P. strobus* , and *P. resinosa* during the autumn (Table 3).

**TABLE 2:** Results from the multilevel model examining the effects of invasion status ( *R. cathartica* invaded vs. *R. cathartica* removed), surrounding human development (Development PC), and season (summer vs. autumn) on the total proportion of seeds removed.

Effect	ϐ	SE	*χ* ^2^	*p*
Invasion	0.07	0.68	0.01	0.910
Development	1.65	5.08	0.10	0.744
Season	−1.81	0.64	7.94	0.004*
Invasion × development	1.95	3.59	0.29	0.586
Invasion × season	−0.65	0.79	0.66	0.413
Season × development	−0.65	3.57	0.03	0.853
Invasion × season × development	−0.96	4.85	0.03	0.842

*Note:* Asterisks indicate significance at a Type I error (α < 0.05).

**TABLE 3:** Random effect coefficients of tree species and small mammal species are represented by the random effect plus the fixed effect estimate to account for the mean slope.

Tree species	Development (rural‐to‐urban)	Season (autumn vs. summer)	Invasion (removed vs. invaded)
*Q. alba*	10.21953	−1.22736	−0.73089
*Q. rubra*	10.91306	−1.23758	−0.80567
*A. saccharum*	−0.42945	−1.83257	0.29337
*P. serotina*	−1.40190	−1.91763	0.38205
*P. strobus*	−3.44220	−2.31515	0.53250
*P. resinosa*	−6.47104	−2.27762	0.85794
Mammal species			
*P. leucopus*	−2.74882	−0.45419	−0.32586
*T. striatus*	−2.33721	−3.46304	−1.30719
*S. carolinensis*	1.51195	0.25203	−0.38183

## 4 Discussion

The spread of invasive species and the expansion of urbanized landscapes, both key agents of global change, can affect both plant and animal communities (Faeth et al. 2005; Fischer et al. 2012; Persons and Eason 2017; Gallo et al. 2019; Guralnick et al. 2020; Hargreaves et al. 2024). Our findings underscore the important role seed traits (i.e., seed mass) play in driving patterns of seed removal by small mammal granivores across a variety of different forest contexts and suggest forest urban contexts may be an important predictor of the removal of certain tree seeds (i.e., *Quercus* spp.). Our work also reinforces the idea that small mammal activity patterns are both species‐specific and structured by multiple, co‐occurring spatial (i.e., urban development, invasive shrub presence) and temporal (i.e., seasonal) environmental variables. Below, we consider how our observed links between novel environments and small mammal activity may help structure tree seed fate in different forest contexts.

### 4.1 Activity of *S. carolinensis* May Negatively Affect Oak Recruitment in Urban Forests

Small mammal granivores may play a significant role in limiting tree establishment from seed in North American forests: large‐scale patterns of regeneration failure of native trees in US forests generate concern regarding the sustainability of this natural resource (Miller et al. 2023), and many reports document the decline of trees in urban landscapes (Catton et al. 2007; Moreira et al. 2018; Piana 2019; Piana et al. 2021). Most studies, however, examine decline using mature trees (e.g., tree responses to herbivory, defoliation, and climatic variation) (Moreira et al. 2018; Romagnoli et al. 2018) rather than factors contributing directly to recruitment limitation (e.g., seed predation, but see Piana 2019). Studies that have examined *Quercus* seed survival and recruitment via *Quercus* seed addition often report low survival because of rodent damage (e.g., 1.0%–7.5% emergence) (Plucinski and Hunter Jr. 2001; Löf et al. 2019; de los Ángeles García‐Hernández and López‐Barrera 2024), and *Quercus* spp. seed survival may be particularly low in urban forests (Conlisk et al. 2012; Piana et al. 2021). Our seed removal results support the idea that oak seed survival is likely to be lowest in urban forests, and our small mammal activity data suggest low *Quercus* seed survival corresponds with greater *S. carolinensis* activity in urban forests. Although implicating *S. carolinensis* as the direct driver of acorn loss in these urban forests will require further examination, the removal of these acorns by rodent seed predators is likely to generate losses in regeneration potential. For example, even if seeds are not immediately consumed, seeds removed by *S. carolinensis* may be stored in arboreal caches where they are unlikely to germinate (Steele et al. 2001, 2011; Bartlow et al. 2018; Sachser et al. 2025). These findings reinforce the need to protect oak acorns during forest management projects and that implementing novel defense techniques (e.g., the application of chemical seed coatings) may be a useful strategy in urban forests (Lanni et al. 2023; Fuka et al. 2024, 2025).

### 4.2 Seasonal *P. leucopus* Activity May Be Shaped by Resource Acquisition

Invasive shrubs have been found to increase small mammal activity by lowering perceived predation risk (Dutra et al. 2011; Bartowitz and Orrock 2016; Guiden and Orrock 2019; Connolly et al. 2024), but changes in resources across seasons may also affect small mammal activity (Dutra et al. 2011). Although the total seed removal was greatest during the summer session (Table 2), we found greater *P. leucopus* activity in the autumn within *R. cathartica* removal plots than in the invaded plots (Figure 1) suggesting that *P. leucopus* may be responding to the presence of supplemental resources provided within seed depots during this season. By creating high‐quality resource patches (i.e., seed depots) for *P. leucopus* in removal plots during resource‐scarce late autumn, the potential costs of anti‐predator behavior may have been outweighed by the introduction of resources (Verdolin 2006; Jacob et al. 2017; Gaynor et al. 2019). These results indicate that rodents are attuned to short‐term changes in habitat quality, thereby modifying their behavior in a dynamic way to match temporal and spatial variability in their environment (Brown and Kotler 2004). It should be noted that the habitat structural complexity to which rodents respond could be a function of both native shrubs and invasive shrubs, but we currently lack studies examining rodent behavioral changes on the basis of native plant cover. These results also imply that quantifying resource availability may be an important aspect of studies examining how invasive shrubs affect animal behavior (e.g., anti‐predator behavior; Mattos et al. 2013; Guiden and Orrock 2019; Stewart et al. 2021). Our results demonstrate that failing to find changes in anti‐predator behavior in invaded habitats may be because animals are responding to pulses of ephemeral resources, not because invasive shrubs are affecting perceived predation risk. Therefore, these findings help to illuminate variation in animal activity and behavior associated with novel environmental changes.

### 4.3 Rodent Preference for Large Seeds Is Not Affected by Invasive Shrubs, Season, or Urbanization

The seeds small mammals choose to consume may reduce the recruitment and establishment of native tree species (Peters et al. 2003; Siepielski and Benkman 2008; Lobo et al. 2009). Rodents often choose seeds by balancing handling time with seed size (Kerley and Erasmus 1991; Muñoz and Bonal 2008; Radtke 2011; Dylewski et al. 2020), and larger seeds are most often associated with high energy content, making them desirable for consumption, caching, or both (Kerley and Erasmus 1991; Steele et al. 2001; Dylewski et al. 2020). Our results are consistent with these findings that seed size was strongly correlated with seed removal, and this trend did not vary on the basis of urbanization, season, or invasion status, further reinforcing the strength of seed size as a predictor of rodent seed removal. Our results mirror other seed removal findings using similar seeds for similar deployment durations (e.g., 95%–100% acorn removal compared to smaller seeds like *P. serotina* and *A. saccharum* ) (Plucinski and Hunter Jr. 2001; Bartowitz and Orrock 2016; Guiden and Orrock 2020; Chandler et al. 2020). Our findings help to identify key seed predators most associated with seed loss by seed mass, specifically suggesting *P. leucopus* and *T. striatus* as primarily consuming smaller seeds like *P. strobus* and *P. resinosa* (Figure 3). In finding a reduction in the removal of smaller seeds during autumn (Table 3), our results suggest that, in addition to small mammals, arthropod seed predators may have been consuming smaller seeded species during the summer (Folgarait and Sala 2002). Although arthropods are prevalent seed predators (Crist and MacMahon 1992; Christian 2001; Ness et al. 2004), they have greater seed size handling restrictions in comparison to rodents (e.g., between 0.26–0.50 mg for ants, Crist and MacMahon 1992; Ness et al. 2004), thereby limiting arthropod seed removal to small‐seeded tree species (Chandler et al. 2020). Our results indicate that rodent body size may affect endotherm food choices, supporting other studies in different systems (Yi et al. 2015). Moreover, Ferreira et al. (2023) examined *Microtus glareolus* seed selection in both risky and safe areas, finding that seed size was the primary predictor of seed removal in riskier areas, further reinforcing our results to demonstrate the importance of seed size in rodent decision making in risky areas.

### 4.4 Conclusions and Future Directions

Given the rapid expansion of urbanized landscapes and the resultant spread of invasive species globally (Nowak and Walton 2005; Shochat et al. 2010; Shifley et al. 2014; Borden and Flory 2021), our results help connect the effects of small mammal activity and native tree survival across a gradient of global change effects. Importantly, the ecological forces structuring urban forests may differ significantly from rural forests (Appendix S2) (Alvey 2006; Piana et al. 2019), and these inherent differences could help to further explain the patterns of seed removal and animal activity we observed. Urban forests are more prone to disturbance, often leading to a community composition dominated by early seral tree species (e.g., *Acer* spp.), whereas more rural forests are ostensibly less intensively disturbed and more likely to host later seral species (e.g., *Quercus* spp.) (Appendix S2, Table S2, and Figure S3). Intrinsic differences in the dominant canopy structure will alter many important habitat features (e.g., vegetation structure, seed rain type and timing), which may alter small mammal community activity on these plots and link back to patterns of tree seed loss. Importantly, because of increased interest in restoring invaded woodlands, developing novel techniques in forest restoration management, specifically centered on protecting native tree seeds from removal and potential consumption, may provide the greatest benefit both in terms of cost‐efficiency and long‐term recruitment success (Löf et al. 2019; Villalobos et al. 2020). Understanding the ways that global change will continue to modify classic plant–animal interactions will be essential to examine in the future to better predict ecological outcomes under different elements of global change.

## Author Contributions

**Mark E. Fuka:** conceptualization (equal), data curation (lead), formal analysis (lead), funding acquisition (supporting), investigation (equal), methodology (equal), resources (equal), validation (equal), visualization (lead), writing – original draft (lead), writing – review and editing (equal). **Brian M. Connolly:** conceptualization (equal), funding acquisition (equal), investigation (equal), methodology (equal), validation (equal), writing – review and editing (equal). **John L. Orrock:** conceptualization (equal), funding acquisition (equal), investigation (equal), methodology (equal), resources (equal), validation (equal), writing – review and editing (equal).

## Conflicts of Interest

The authors declare no conflicts of interest.

## Supporting information


**Data S1:** ece372038‐sup‐0001‐DataS1.docx.

## Data Availability

All data analyzed and corresponding code in this article can be found on the online data repository site FigShare: https://figshare.com/s/a31dcc8ef56ea0605ae1.
