# Supplementary material for: Urbanization, Not Invasive Shrubs, Alters Tree Seed Fate by Modifying Rodent Activity
Source: Ecol Evol. 2025 Aug 22;15(8):e72038. doi: 10.1002/ece3.72038 (PMC12373979; doi:10.1002/ece3.72038)
Supplement: Supplementary file 1 — Data S1: ece372038‐sup‐0001‐DataS1.docx. [file ECE3-15-e72038-s001.docx]

**Appendices**

Appendix S1

*Site selection*

To select forested sites, we visited and surveyed more than 170 forested sites in southern Wisconsin during the summer and autumn of 2021. In collaboration with the University of Wisconsin System, Wisconsin Department of Natural Resources (W-DNR), various county and city parks departments, local school districts and nonprofits, we were able to identify sites that adhered to our criteria. Site level assessments of topography, fire history, flooding, density and composition of tree canopies, density of invasive shrub canopies, forest stand size, and adjacent land use were surveyed. Sites were chosen that had no noticeable history of fire or seasonal flooding. Importantly, sites were chosen with deciduous hardwood canopies that were dense enough so that subsequent invasive shrub removal would not render plots as savannas or open canopy sites. Lastly, sites were large enough to contain our two, 400m^2^ plots with a 20m distance from a hard edge. In total, our site selection efforts produced 15 sites across approximately 10,000 km^2^ in south central Wisconsin (Figure S2).

*Site classification*

An important term used in our analyses focused on the varying levels of human development surrounding each forested site. To classify forests along a rural-to-urban gradient for use in analysis, landcover data downloaded from the National Land Cover Database (NLCD) was used to create a principal component analysis to examine the variation in landcover surrounding each site. We used the sf and raster packages (Pebesma and Bivard 2023; Hijames 2025) in R (R Core Team 2024) to create a 250 buffer around each forest polygon. We then used the cdlTools package (USDA-NASS 2024; Chen et al. 2024) to download the 2021 USDA cropland data layer for Wisconsin. We categorized each landcover type in the data layer as either agricultural, forest, development, wetland, water, or other (Table S1). We then used the buffer to calculate the proportion of each category surrounding the polygon in the 250m buffer. We then checked these proportions against the site classification assigned during the site visit to ensure a match. The results of the PCA revealed that negative values were associated with more rural sites and positive values were associated with more urban sites. Additionally, the first Development PC axis (PC1) explained 70.18% of the variation between sites along this gradient while the second Development PC axis (PC2) explained only 29.82%. When plotting sites using both Development PC axes, sites fall along an urbanization gradient where most of the variation is explained by Development PC axis 1 (PC1) (Figure S1).

**Tables**

Table S1. Output from the surrounding 250m landcover principal component analysis.

|  |  |  |  |
| --- | --- | --- | --- |
|  | PC1 | PC2 | PC3 |
| Agriculture | -0.077 | -0.469 | 0.631 |
| Development | 0.816 | 0.342 | -0.056 |
| Forest | -0.559 | 0.641 | -0.086 |
| Open Water | -0.007 | -0.019 | 0.056 |
| Wetland | -0.086 | -0.500 | -0.740 |
| Other | -0.085 | 0.00 | 0.196 |

**Figures**


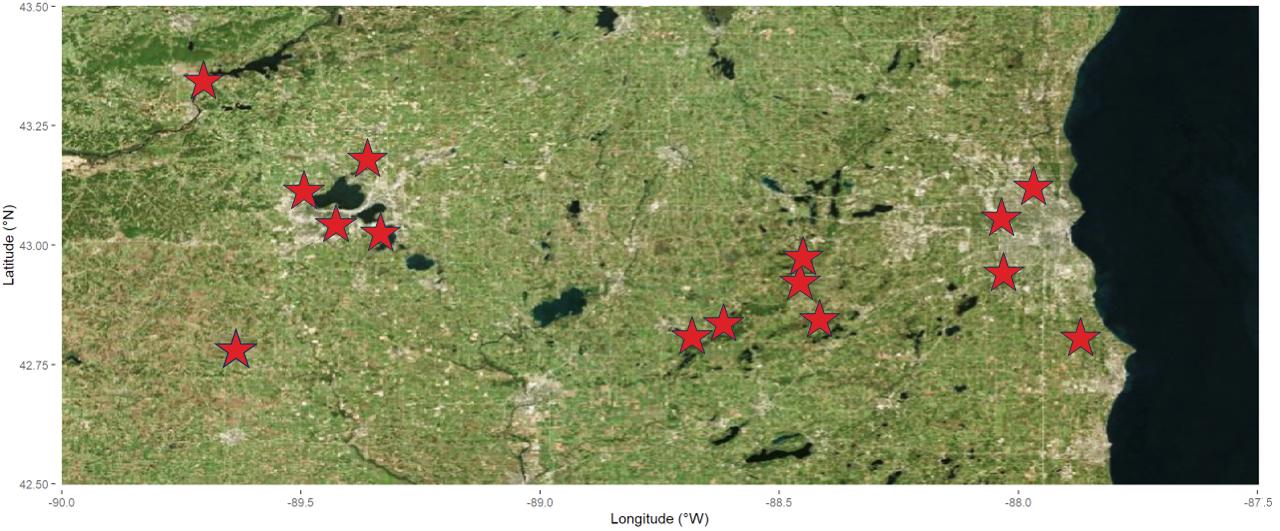


Figure S1. Map depicting forested sites across southern and southeastern Wisconsin (U.S.A).


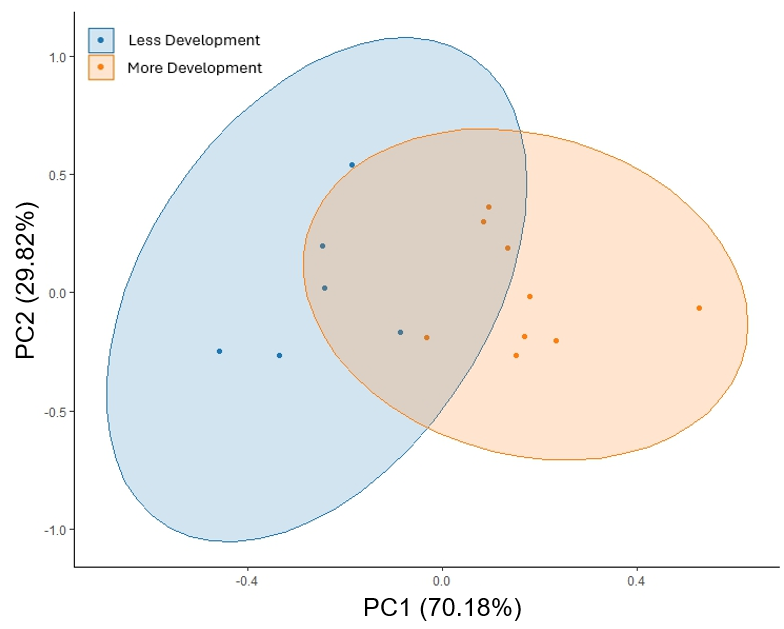


Figure S2. Overlap plot of each site based on Development PC axes 1 and 2 split by more vs. less development.

**References**

Chen, L., Lisic, J., Stachelek, J. 2024. Tools to Download and Work with USDA Cropscape Data. R package version 0.14. <https://cran.r-project.org/package=cdlTools>

Hijmans R 2025. raster: Geographic Data Analysis and Modeling_. R package version 3.6-32, <https://CRAN.R-project.org/package=raster>.

Pebesma, E., & Bivand, R. 2023. Spatial Data Science: With Applications in R. Chapman and Hall/CRC. <https://doi.org/10.1201/9780429459016>

R Core Team 2024. R: A Language and Environment for Statistical Computing_. R Foundation for Statistical Computing, Vienna, Austria. <https://www.R-project.org/>.

USDA-NASS. 2024 National Agricultural Statistics Service Cropland Data Layer. https://nassgeodata.gmu.edu/CropScape/

Appendix S2

We measured several aspects of the environment that may be fundamentally different in highly urban forests compared to rural forests. We used generalized linear mixed effects models to examine differences in plot level overstory tree canopy composition (measured summer 2022), ambient aboveground temperature (measured spring 2023), soil compaction (measured summer 2022) and soil moisture holding capacity (measured spring 2024). Each model included habitat invasion (*R. cathartica* invaded vs. *R. cathartica* removed), human development (Development PC) and their interaction with site as a random effect.

**Table**

Table S2.

Results from generalized linear mixed effects models of the effects that habitat invasion (*R. cathartica* invaded vs. *R. cathartica* removed) and human development (Development PC) have on site level tree canopy composition, ambient temperature, soil compaction, and soil moisture holding capacity.

|  |  |  |  |  |  |  |
| --- | --- | --- | --- | --- | --- | --- |
| Tree Canopy Composition |  |  |  | Soil Compaction |  |  |
| Effect | *χ2-value* | *p-value* |  | Effect | *χ2-value* | *p-value* |
| Invasion | 0.01 | 0.924 |  | Invasion | 1.73 | 0.187 |
| Development | 192.81 | **< 0.001** |  | Development | 1.16 | 0.281 |
| Habitat x Development | 0.59 | 0.44 |  | Habitat x Development | 0.22 | 0.631 |
|  |  |  |  |  |  |  |
| Ambient Temperature |  |  |  | Soil Moisture Holding Capacity |  |  |
| Effect | *χ2-value* | *p-value* |  | Effect | *χ2-value* | *p-value* |
| Invasion | 0.01 | 0.921 |  | Invasion | 0.58 | 0.443 |
| Development | 2.88 | *0.089* |  | Development | 0.39 | 0.529 |
| Habitat x Development | 0.05 | 0.811 |  | Habitat x Development | 0.32 | 0.568 |


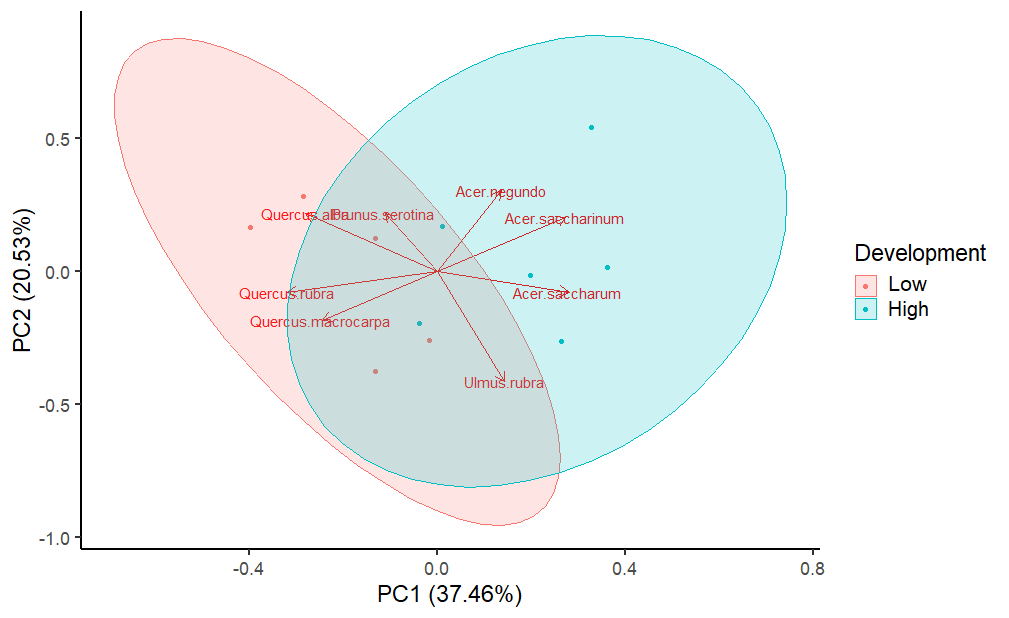
**Figures**

Figure S3. Overlap plot of each site based on Tree Composition PC axes 1 and 2 split by low vs. high human development.
